# Global research on RNA vaccines for COVID-19 from 2019 to 2023: a bibliometric analysis

**DOI:** 10.3389/fimmu.2024.1259788

**Published:** 2024-02-15

**Authors:** Ziyi Chen, Zhiliang Liu, Yali Feng, Aochen Shi, Liqing Wu, Yi Sang, Chenxi Li

**Affiliations:** ^1^ Center for Molecular Diagnosis and Precision Medicine, The First Affiliated Hospital, Jiangxi Medical College, Nanchang University, Nanchang, China; ^2^ Jiangxi Key Laboratory of Cancer Metastasis and Precision Treatment, the First Hospital of Nanchang, Nanchang, China; ^3^ Department of Pathology, Jiangxi Cancer Hospital, Nanchang, China; ^4^ Department of Pathology, Jiangxi Provincial Chest Hospital, Nanchang, China

**Keywords:** COVID-19, SARS-CoV-2, RNA vaccines, web of science, bibliometrics

## Abstract

**Background:**

Since the global pandemic of COVID-19 has broken out, thousands of pieces of literature on COVID-19 RNA vaccines have been published in various journals. The overall measurement and analysis of RNA vaccines for COVID-19, with the help of sophisticated mathematical tools, could provide deep insights into global research performance and the collaborative architectural structure within the scientific community of COVID-19 mRNA vaccines. In this bibliometric analysis, we aim to determine the extent of the scientific output related to COVID-19 RNA vaccines between 2019 and 2023.

**Methods:**

We applied the Bibliometrix R package for comprehensive science mapping analysis of extensive bibliographic metadata retrieved from the Web of Science Core Collection database. On January 11th, 2024, the Web of Science database was searched for COVID-19 RNA vaccine-related publications using predetermined search keywords with specific restrictions. Bradford’s law was applied to evaluate the core journals in this field. The data was analyzed with various bibliometric indicators using the Bibliometrix R package.

**Results:**

The final analysis included 2962 publications published between 2020 and 2023 while there is no related publication in 2019. The most productive year was 2022. The most relevant leading authors in terms of publications were Ugur Sahin and Pei-Yong, Shi, who had the highest total citations in this field. The core journals were Vaccines, Frontiers in Immunology, and Viruses-Basel. The most frequently used author’s keywords were COVID-19, SARS-CoV-2, and vaccine. Recent COVID-19 RNA vaccine-related topics included mental health, COVID-19 vaccines in humans, people, and the pandemic. Harvard University was the top-ranked institution. The leading country in terms of publications, citations, corresponding author country, and international collaboration was the United States. The United States had the most robust collaboration with China.

**Conclusion:**

The research hotspots include COVID-19 vaccines and the pandemic in people. We identified international collaboration and research expenditure strongly associated with COVID-19 vaccine research productivity. Researchers’ collaboration among developed countries should be extended to low-income countries to expand COVID-19 vaccine-related research and understanding.

## Introduction

Since 2019, the global COVID-19 pandemic has affected the lives of billions of people worldwide (1). To deal with this situation, countries worldwide began to develop vaccines, including traditional inactivated vaccines, recombinant protein, live-attenuated vaccines, RNA vaccines, etc. (2–15). On October 2nd, 2023, the Nobel Assembly at the Karolinska Institutet decided to award the 2023 Nobel Prize in Physiology or Medicine jointly to Katalin Karikó and Drew Weissman for their discovery of nucleoside base modifications, which made it possible to develop an effective mRNA vaccine against COVID-19 (16). RNA vaccines have received widespread attention due to their high efficacy, specificity, versatility, rapid and large-scale development capabilities, low-cost production potential, and safety (17, 18). RNA vaccines have been developed for several decades (19, 20), and since COVID-19 has broken worldly, the RNA vaccines platform has enabled fast vaccine development in response to this pandemic (21). RNA vaccines provide flexibility in the design and expression of vaccine antigens, simulating the structure and expression of antigens during natural infections. RNA is necessary for protein synthesis and unconformity into the genome, and it is transiently expressed, metabolized, and eliminated by the body’s natural mechanism (22), so it is considered relatively safe. Many clinical trials have proven RNA-based preventive infectious disease vaccines and RNA therapeutic agents to be safe and well-tolerated (23–29). Generally speaking, vaccination with RNA can trigger a robust innate immune response. RNA guides the expression of vaccine antigens in host cells and has intrinsic adjuvant effects (30–32). One advantage of the RNA vaccine manufacturing platform is that it can quickly produce many vaccines targeting new pathogens, regardless of the encoded pathogen antigen (33). The bibliometric analysis of published articles provides insights into research prospects, gaps, and future directions in the research field. This study examined scientific publications related to RNA vaccines for COVID-19 through bibliometric analysis and trend analysis.

## Results

### Search strategy

**Figure 1:**
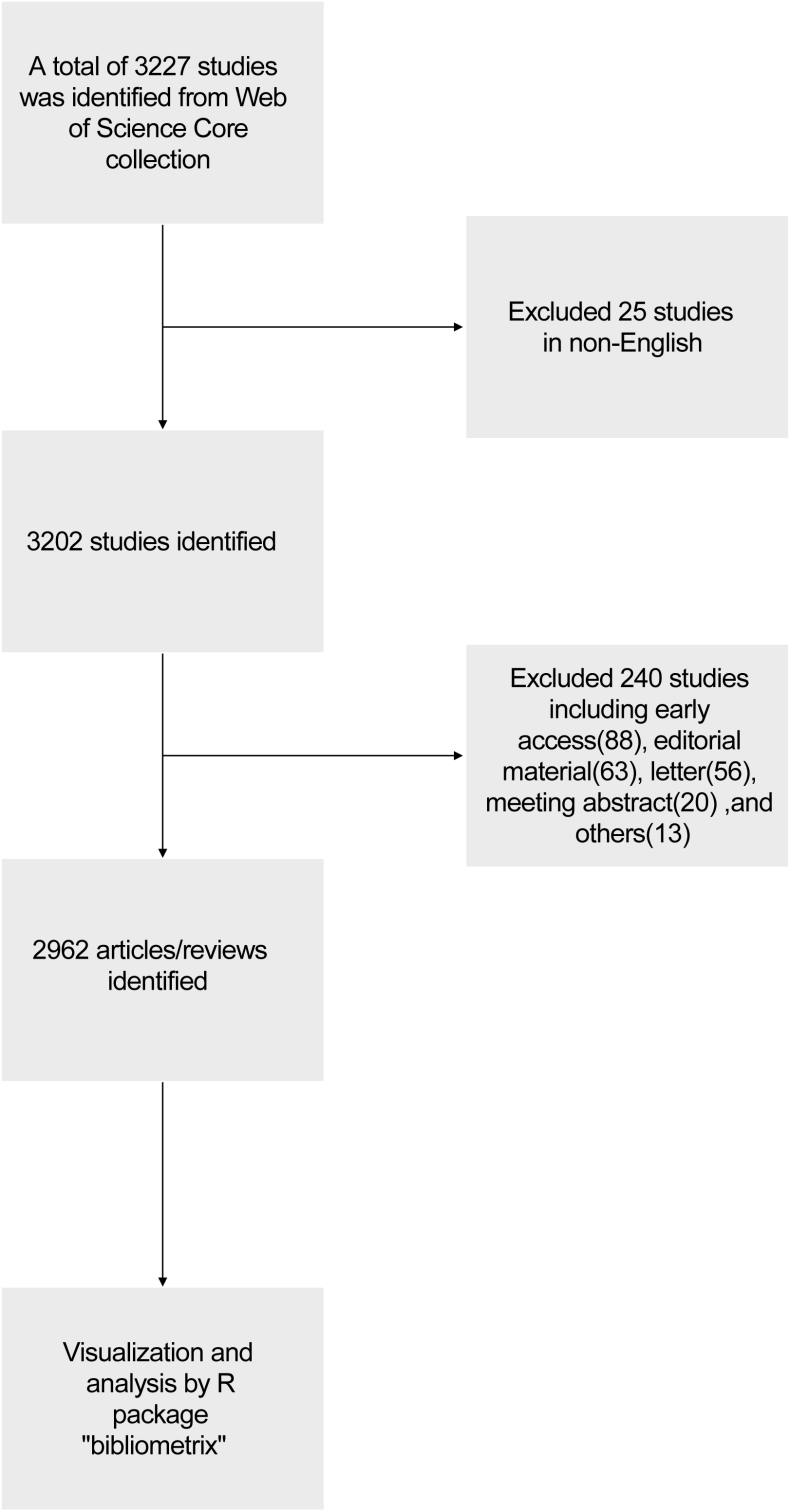
Flowchart of the related data collection and bibliometrics analysis.

We conducted a literature search on the Web of Science Core Collection (WoSCC) database (https://www.webofscience.com/wos/woscc/basic-search) on January 11th, 2024. The search formula was TS= ((RNA vaccine AND COVID-19) OR (RNA vaccine AND SARS-COV-2)), the published year was set before 2024, and the type of documents was set to articles and reviews. The language filter was set in English (**Figure 1**). According to our search strategies, there were 2962 studies of RNA vaccines for COVID-19 between 2020 and 2023 (0 publication in 2019), including 1956 articles and 1006 reviews. The analyzed publications were written by 23141 authors (93 with single-authored documents and 23048 with multi-authored documents) from 104 countries and 908 journals.

### Characteristics of the year of publication

**Figure 2:**
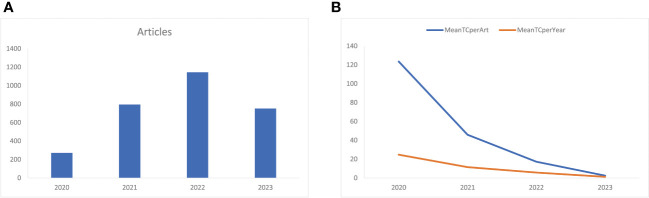
**(A) **Annual related publication from 2019 to 2023 per year, and **(B)** average article and average article citations from 2019 to 2023 in COVID-19 RNA vaccine-related research. MeanTCperArt, mean total citation per article; MeanTCperYear, mean total citation per year.

**Figure 2A** shows that the number of annual related publications increased rapidly year by year from 2020 to 2022. In 2020, 271 articles were published, while 795 in 2021, 1144 in 2022, and 752 in 2023. The most productive year was 2022 (n = 1144) with the annual scientific growth rate of 143.9%. The total number of citations per article and the average citations per year have decreased (**Figure 2B**). In 2020, the average number of citations per article was 123.5, 45.9 in 2021, 17.3 in 2022, and 2.48 in 2023. The total average number of citations per year was 24.7 in 2020, 11.5 in 2021, 5.8 in 2022, and 1.2 in 2023.

### Characteristics of the countries

**Figure 3:**
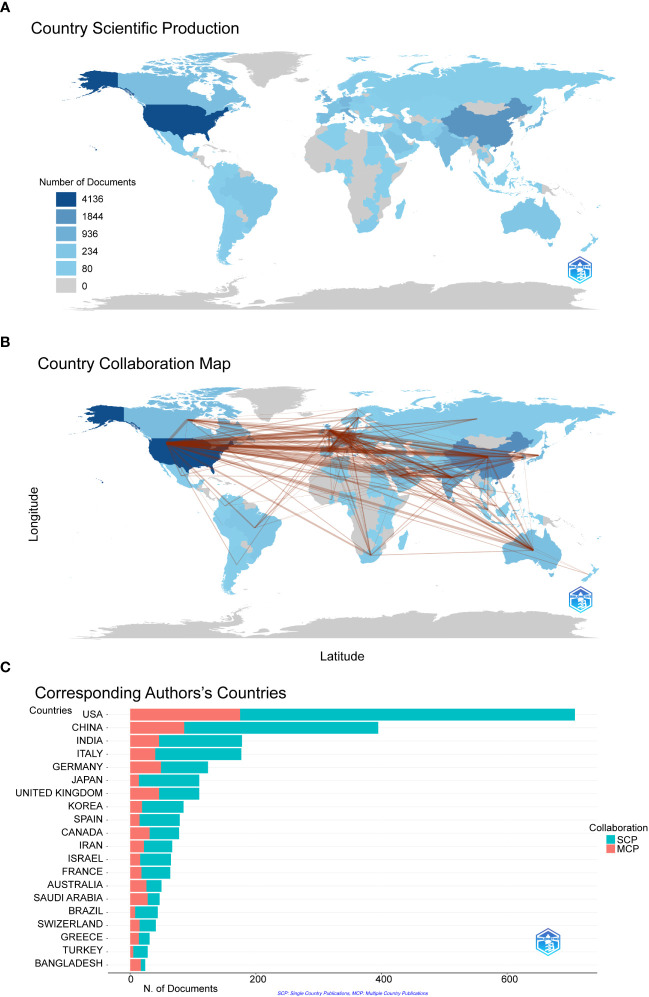
The geographical distribution **(A)** and visualization **(B)** of countries on the research of COVID-19 RNA vaccines. A choropleth map detailing the geographic distribution of collaborating countries. The intensity (from light blue to dark blue) is proportional to the number of publications. The number of links (presented as red lines) between any two countries represents the strength of collaboration. **(C)** Co-authorship analysis of countries in the related research SCP (Single country publications) indicates that the authors of this article are all from the same country, and MCP (Multiple country publications) indicates that the authors of this article are from different countries, indicating international cooperation.

We filtered and visualized 104 countries that published more than ten articles and constructed a collaborative network based on the number and relationship of publications in each country. From **Figure 3A**, we can point out that the United States has the highest literature output(n=4163) on COVID-19 RNA vaccines, and the number is significantly higher than that of China(n=1844) and Italy(n=936). Notably, there is much active cooperation between different countries. For example, the United States closely cooperates with China, the United Kingdom, Germany, and Italy; India actively cooperates with Saudi Arabia (**Figure 3B**). It shows that the United States has the most significant number of SCPs and MCPs, which indicates that the United States has the most researches on COVID-19 RNA vaccines and cooperation with other countries in this regard, followed by China on both SCP and MCP (**Figure 3C**).

### Characteristics of the affiliations

**Figure 4:**
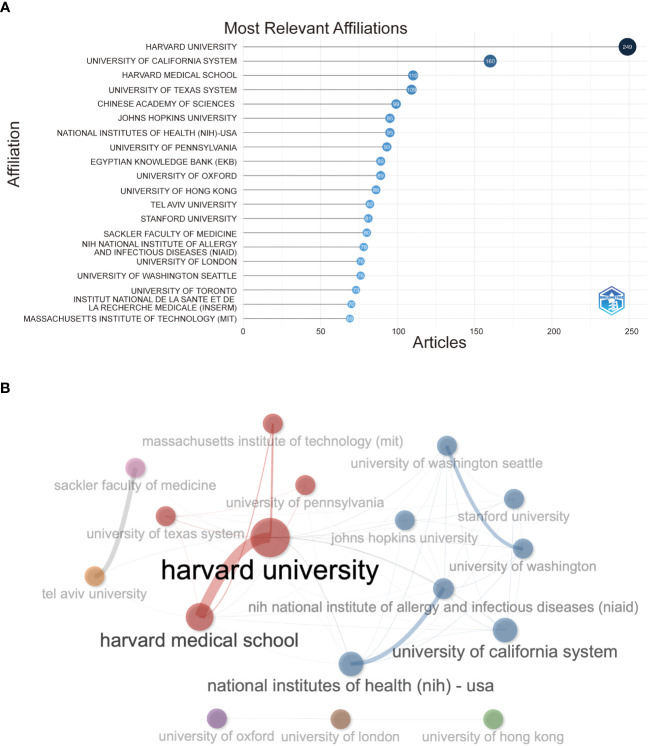
**(A)** Top 20 most relevant affiliations on the research of COVID-19 RNA vaccines. **(B) **Network map of co-authorship between affiliations with more than 5 citations.

In **Figure 4A**, Harvard University has the highest number of institutions that receive and publish articles (n=249), followed by the University of California System (n=160) and Harvard Medical School (n=76). Half the top 20 most relevant affiliations were from the United States, followed by the United Kingdom, China, France, and Israel. Subsequently, we selected 34 institutions based on visualization with a minimum number of publications equal to 5. We constructed a collaborative network based on the number of publications and relationships of each affiliation (**Figure 4B**). As shown in **Figure 4B**, Harvard University and Harvard Medical School cooperated the most, and Tel Aviv University and Sheba Medical Center also had active cooperation. In addition, we noticed that Harvard University had published the most papers and collaborated with the most significant number of affiliations.

### Characteristics of the top 20 most productive authors

The number of academic publications by an author can represent research activities and contributions in the field to some extent. As shown in **Table 1**, Ugur Sahin was the most influential author from University Medical Center, Johannes Gutenberg University, between 2020 and 2023 on COVID-19 RNA vaccines, who had published 14 articles in this field, whose h-index is 9, g-index is 14, m-index is 2.6. He also has the highest number of total citations(n=14203). Pei-Yong Shi’s h-index(n=12) is a close second. Pei-Yong Shi published 16 articles in this field between 2020 and 2023; his g-index is 16, and his m-index is 2.4. Notably, we can find that Pei-Yong Shi and Ugur Sahin had the most significant academic influence on COVID-19 RNA vaccines.

**Table 1:** The author’s impact in relevant field.

Element	h_index	g_index	m_index	TC	NP	PY_start
SAHIN U	13	14	2.6	14203	14	2020
SHI PY	12	16	2.4	4269	16	2020
LIU Y	10	16	2.0	502	16	2020
TÜRECI Ö	10	10	2.0	13602	10	2020
LEE J	9	17	1.8	305	17	2020
LI X	9	14	1.8	383	14	2020
LUSTIG Y	9	12	2.3	1277	12	2021
CHEN Y	8	13	1.6	644	13	2020
DORMITZER PR	8	8	1.6	13681	8	2020
KUMAR A	8	13	1.6	335	13	2020
SINGH S	8	12	2.0	296	12	2021
SWANSON KA	8	8	1.6	13847	8	2020
ZHANG Y	8	15	2.0	511	15	2021
BARIC RS	7	9	1.4	908	9	2020
CHAN EWY	7	7	2.3	193	7	2022
CHUI CSL	7	7	2.333	193	7	2022
GAO GF	7	8	1.4	338	8	2020
KIM J	7	15	1.75	588	15	2021
KUMAR S	7	13	1.4	330	13	2020
LAI FTT	7	7	2.333	193	7	2022

H-index means a scientist has published at least h papers, whose citation frequency is no less than h, within a certain period. G-index means that the maximum paper order g with a cumulative citation of at least g square times, which is ranked relatively high by the number of citations, means that the cumulative citation number corresponding to the (g+1) th order paper will be less than the square of (g+1). M-index means h-index divided by the author’s academic age.

### Characteristics of the top 20 journals and co-cited journals

**Figure 5:**
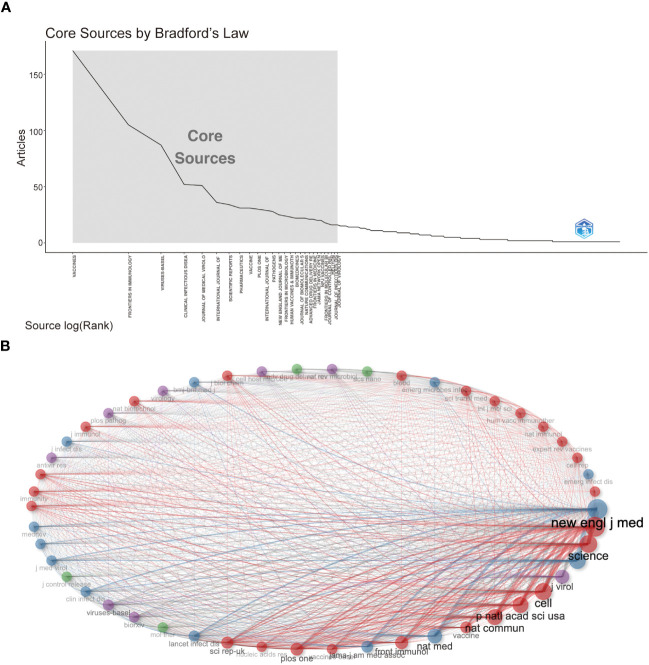
**(A) **Journals (Sources) clustering through Bradford’s law. **(B)** Co-cited Journals of COVID-19 RNA vaccines.

Followed by Frontiers in Immunology (n=105, 3.54%) and Viruses-Basel (n = 58, 2.94%), the Vaccines published the most articles on COVID-19 RNA vaccines (n =171, 5.77%) throughout four years. However, the New England Journal of Medicine, Nature, and Science were the most cited journals. Bradford’s law was applied to assess the core journals in the field of COVID-19 RNA vaccines. As shown in **Figure 5A**, the core journals in COVID-19 RNA vaccines were Vaccines, Frontiers in Immunology, Viruses-Basel, Clinical Infectious Diseases, Journal of Medical Virology, etc. As for co-cited journals in **Figure 5B**, journals were categorized into different clusters. The nodes with different colors in the graph represent different clusters. The node size represents the number of articles published in the journal, and the thickness of the lines represents the number of connections between nodes. Frontiers in Immunology, Vaccines, and Journal of Medical Virology were the top three most influential journals in this field. This result can help scholars to select the best-fit journals for submitting their research findings. Also, **Table 2** lists the top 20 most-cited publications on COVID-19 RNA vaccines. All these productions were published between 2020 and 2023, and 65% obtained more than 1000 citations. **Table 2** shows that the New England Journal of Medicine was the highest-cited journal with the highest h-index, m-index, and total citations. Frontiers in Immunology has the highest g-index. These indexes showed the importance of these two journals on COVID-19 RNA vaccines.

**Table 2:** The journal’s impact on COVID-19 RNA vaccines.

Journal	h_index	g_index	m_index	Total citations	Number of publications	Starting Publishing year
**NEW ENGLAND JOURNAL OF MEDICINE**	24	25	4.8	17419	25	2020
**NATURE**	9	9	1.8	4109	9	2020
**SCIENCE**	13	14	3.25	2938	14	2021
**NATURE MEDICINE**	13	14	3.25	2393	14	2021
**VACCINES**	22	40	4.4	2100	171	2020
**FRONTIERS IN IMMUNOLOGY**	18	43	3.6	2015	105	2020
**CELL**	8	9	1.6	1619	9	2020
**JOURNAL OF MEDICAL VIROLOGY**	17	37	3.4	1390	51	2020
**VIRUSES-BASEL**	19	34	3.8	1256	87	2020
**NATURE REVIEWS IMMUNOLOGY**	6	6	1.2	1190	6	2020
**NATURE REVIEWS MATERIALS**	2	2	0.4	1140	2	2020
**ACS CENTRAL SCIENCE**	3	3	0.6	1131	3	2020
**ADVANCED DRUG DELIVERY REVIEWS**	14	21	3.5	1073	21	2021
**NATURE COMMUNICATIONS**	14	22	2.8	995	22	2020
**CLINICAL INFECTIOUS DISEASES**	16	29	4	939	52	2021
**ANNALS OF THE RHEUMATIC DISEASES**	7	7	1.75	854	7	2021
**JAMA CARDIOLOGY**	5	5	1.25	843	5	2021
**JAMA-JOURNAL OF THE AMERICAN MEDICAL ASSOCIATION**	5	5	1.25	793	5	2021

### Relations between journals (left), authors (middle), and affiliations(right)

**Figure 6:**
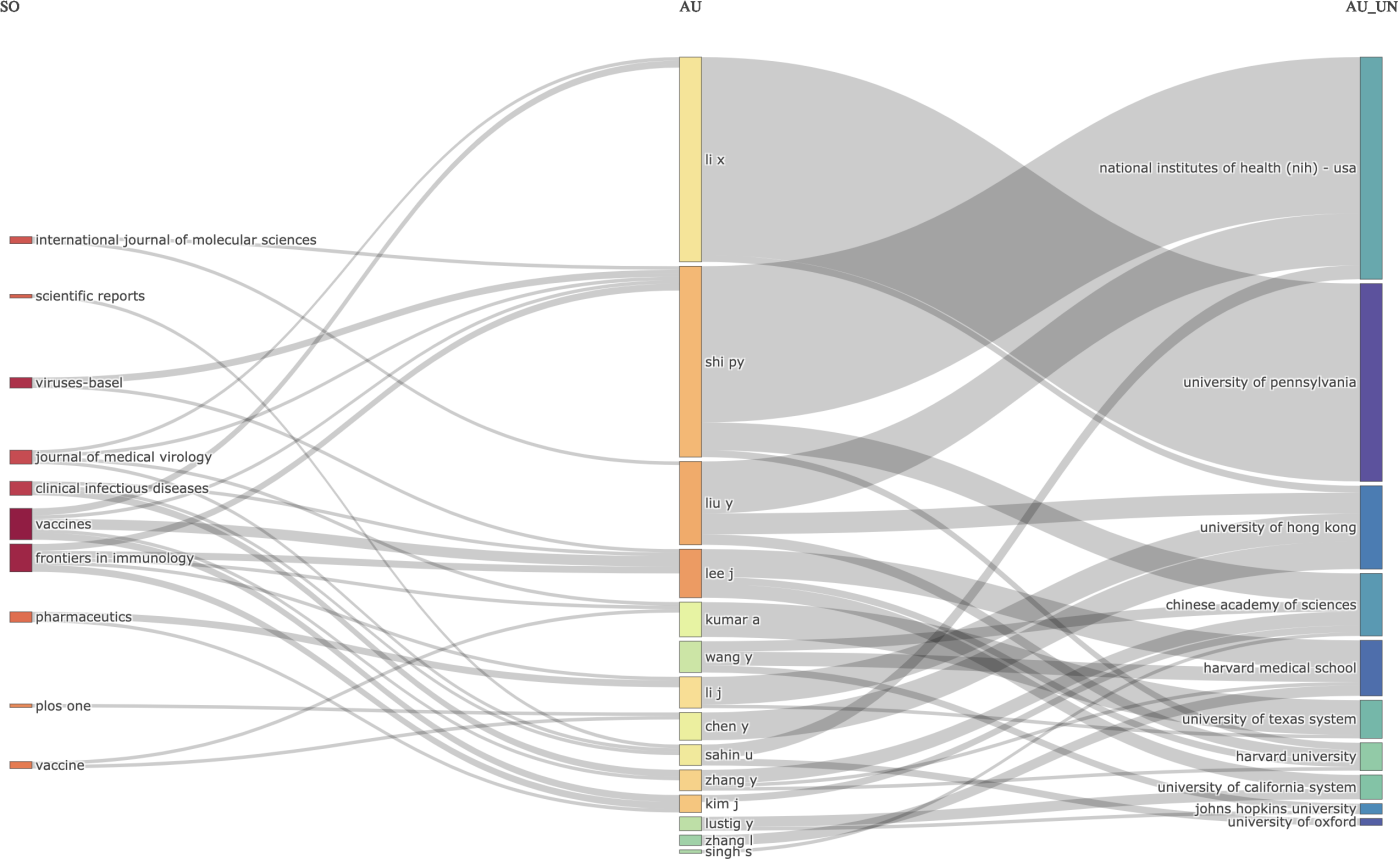
Three-Fields Plot revealed the relations between journals (left), authors (middle), and affiliations (right) for research in COVID-19 RNA vaccines.

The relations between journals, authors, and affiliations were visualized using the three-field plot (TFP). In this instance, the significant features were represented in the diagram by rectangles with different colors. The height of the rectangles in the diagram of the TFP depended on the rate or value of the summation of the relations arising between the component the rectangle represents (journals, authors, and affiliations) and the diagram of other elements. The more relations the component or element had, the higher the rectangle represented. **Figure 6** shows the TFP analysis of publications on COVID-19 RNA vaccines centered on relations between the journals, authors, and affiliations. The diagram demonstrated the top journals, authors, and affiliations relations in publications on COVID-19 RNA vaccines and their related studies during these four years.

### Characteristics of the top 20-most cited articles and co-cited references

The top 20 most cited articles were published in 11 journals between 2020 and 2023 (**Table 3**). Seven articles were published in The New England Journal of Medicine, and four were published in Nature. With 8609 citations, the top-cited article was published by Fernando P Polack from the New England Journal of Medicine in 2020. The total citations per year were 1721.80, and the normalized total citation was 69.69. The following one was published by Edward E Walsh and received 1574 citations, whose total citations per year was 314.80, and the normalized total citation was 12.74.

**Table 3:** Main characteristics of the top 20-most cited articles.

NO.	Articles	Journals	IF	First Author	Total Citations (TC)	TC per Year	Normalized TC.	Publication Year
1	Safety and Efficacy of the BNT162b2 mRNA Covid-19 Vaccine	NEW ENGL J MED	158.5	POLACK FP	8609	1721.8	69.69	2020
2	Safety and Immunogenicity of Two RNA-Based Covid-19 Vaccine Candidates	NEW ENGL J MED	158.5	WALSH EE	1574	314.8	12.74	2020
3	COVID-19 vaccine BNT162b1 elicits human antibody and TH1 T cell responses	NATURE	64.8	SAHIN U	1070	214.0	8.66	2020
4	Safety and Immunogenicity of SARS-CoV-2 mRNA-1273 Vaccine in Older Adults	NEW ENGL J MED	158.5	ANDERSON EJ	959	191.8	7.76	2020
5	Phase I/II study of COVID-19 RNA vaccine BNT162b1 in adults	NATURE	64.8	MULLIGAN MJ	940	188.0	7.61	2020
6	Lipid nanoparticles for mRNA delivery	NAT REV MATER	83.5	HOU XC	938	234.5	20.44	2021
7	Covid-19 Breakthrough Infections in Vaccinated Health Care Workers	NEW ENGL J MED	158.5	BERGWERK M	886	221.5	19.31	2021
8	Research and Development on Therapeutic Agents and Vaccines for COVID-19 and Related Human Coronavirus Diseases	ACS CENTRAL SCI	18.2	LIU C	826	165.2	6.69	2020
9	Pathogenesis and transmission of SARS-CoV-2 in golden hamsters	NATURE	64.8	SIA SF	823	164.6	6.66	2020
10	Protection of BNT162b2 Vaccine Booster against Covid-19 in Israel	NEW ENGL J MED	158.5	BAR-ON YM	764	191.0	16.65	2021
11	Safety and Efficacy of the BNT162b2 mRNA Covid-19 Vaccine through 6 Months	NEW ENGL J MED	158.5	THOMAS SJ	710	177.5	15.47	2021
12	Potential Interventions for novel coronavirus in China: A systematic review	J MED VIROL	12.7	ZHANG L	644	128.8	5.21	2020
13	Coronavirus Infections in Children Including COVID-19: An Overview of the Epidemiology, Clinical Features, Diagnosis, Treatment and Prevention Options in Children	PEDIATR INFECT DIS J	3.6	ZIMMERMANN P	641	128.2	5.19	2020
14	Immunological Considerations for COVID-19 Vaccine Strategies	NAT REV IMMUNOL	100.3	JEYANATHAN M	612	122.4	4.95	2020
15	Infection and Rapid Transmission of SARS-CoV-2 in Ferrets	CELL HOST MICROBE	30.3	KIM YI	603	120.6	4.88	2020
16	Safety of the BNT162b2 mRNA Covid-19 Vaccine in a Nationwide Setting	NEW ENGL J MED	158.5	BARDA N	596	149.0	12.99	2021
17	Cutaneous reactions reported after Moderna and Pfizer COVID-19 vaccination: A registry-based study of 414 cases	J AM ACAD DERMATO	13.8	MCMAHON DE	516	129.0	11.24	2021
18	Altered TMPRSS2 usage by SARS-CoV-2 Omicron impacts infectivity and fusogenicity	NATURE	60.9	MENG B	502	167.3	29.08	2022
19	Efficacy of the BNT162b2 mRNA COVID-19 Vaccine in Patients with Chronic Lymphocytic Leukemia	BLOOD	20.3	HERISHANU Y	450	112.5	9.81	2021
20	Immune Cell Profiling of COVID-19 Patients in the Recovery Stage by Single-Cell Sequencing	CELL DISCOV	33.5	WEN W	426	85.2	3.45	2020

**Figure 7:**
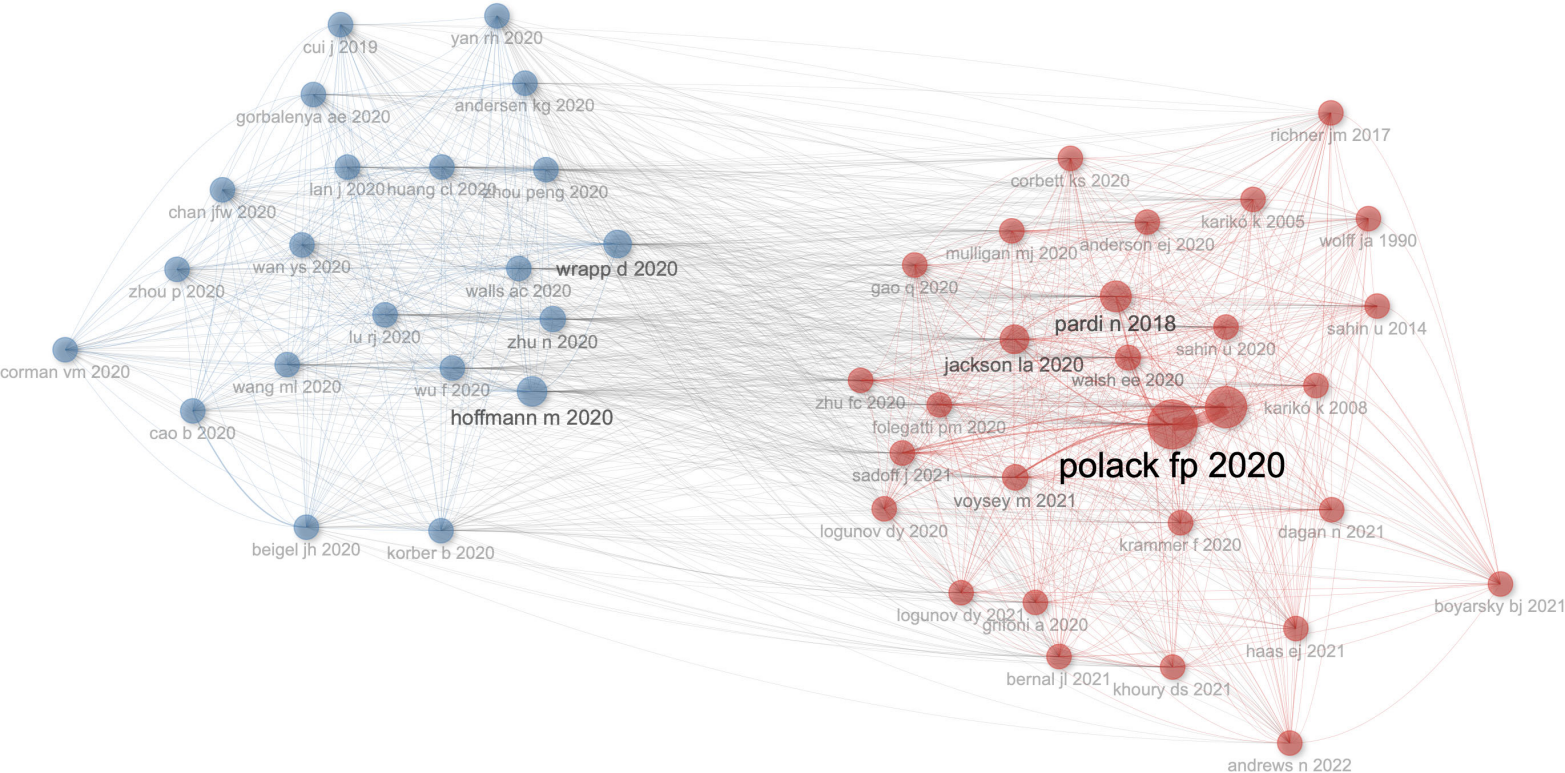
Network map of co-citation between references with more than five citations.

There are 50 references of co-citation with more than five citations. As shown in **Figure 7**, Wrapp d 2020 (34) has the highest number of connections with other references, followed by Hoffmann m 2020 (35). Polack fp 2020 (24) has the highest value of PageRank to get other references, which shows the importance of a node to get other nodes, followed by Baden lr 2021 (36).

### Keyword co-occurrence, clusters

**Figure 8:**
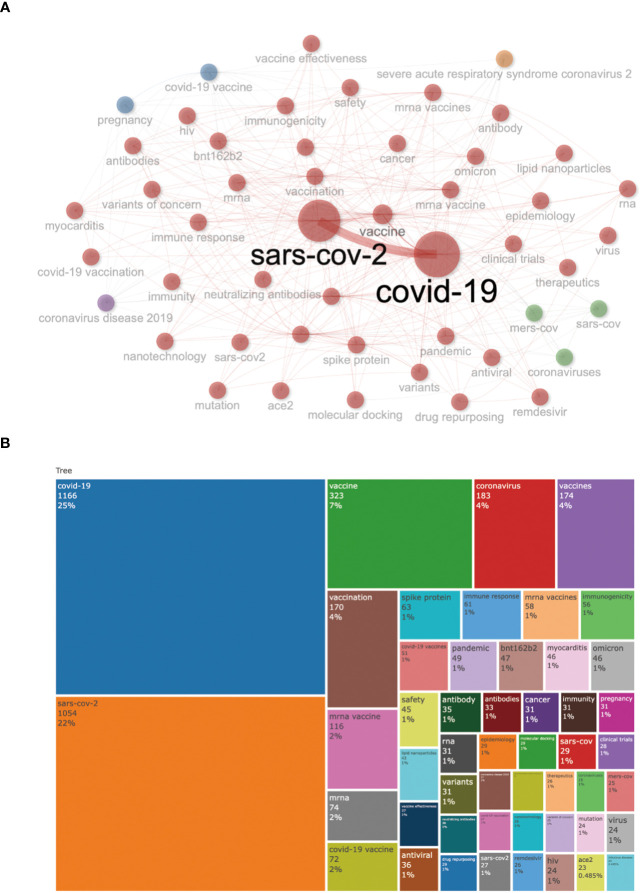
Keyword co-occurrence map **(A)** and the cluster of COVID-19 RNA vaccines **(B)**.

Keywords are always the core research content highly condensed and summarized by researchers, which can reflect the central theme of the research. Therefore, keyword co-occurrence analysis is a crucial way to determine the main research direction and hot research topics of a specific discipline. Among **Figure 8B**, the most frequent author’s keywords were “covid-19” (n =1166,25%), “sars-cov-2” (n=1054,22%), “vaccine” (n=323,7%), “coronavirus” (n =183,4%), “vaccines” (n =174,4%), and “vaccination” (n = 170,4%). The overall keyword network visualization is presented in **Figure 8**. It can be seen that the frequency of the words COVID-19 and SARS-COV-2 has significantly increased from 2020 to 2023.

## Discussion

In this study, R studio quantitative analysis software was used to analyze the references related to the COVID-19 RNA vaccines and summarize the research results and progress. Quantitative analysis of annual publication quantity, country, author, institution, journal, and other essential information are also included. According to the number of articles published on the COVID-19 RNA vaccines in 2020, the number of documents published in this field is 271, showing an overall increasing trend. The higher the number of citations in a paper, the more excellent its impact on the field and the higher its quality. The total number of citations in this field increases between 2020 and 2022. The number of related articles in 2023 is lower than in 2022.

Through statistical analysis of the number of papers published by countries/regions and institutions, it can be determined that the key countries/regions and research institutions that have published many COVID-19 RNA vaccine literature and have a significant influence can determine their cooperation relationship. The United States and China are major countries conducting research on RNA vaccines for COVID-19, and the United States ranks first. Half of the top 20 research institutions are in the United States, followed by the United Kingdom, China, France and Israel. We noticed the close cooperation among five countries: the United States, China, the United Kingdom, Germany, and Italy. In addition, the United States has active collaborations with China, the United Kingdom, and Germany. The United States is undoubtedly the main driving force for the development of this field. The publications and cooperation between countries are significantly higher in developed countries than in developing countries. Regarding research affiliations, 50% of the top 20 most relevant affiliations were from the United States, which may be one of the essential reasons for the rapid development of the United States in this field. Regarding institutions, Harvard University is the most prolific institution, followed by the University of California System and Harvard Medical School. Affiliations like Tel Aviv University and Sheba Medical Center have an excellent cooperative relationship. Also, we found that Harvard University published the most papers and collaborated with the most institutions, which will be detrimental to the long-term development of academic research. Although some countries have cooperative relations, the frequency, breadth, and intensity of cooperation between institutions are not ideal. For example, there is only a small amount of collaboration between institutions in the United States and China. This situation will hinder the development of the research field in the long run. Therefore, we strongly recommend that research institutions in various countries carry out extensive cooperation and communication to jointly promote the development of RNA vaccines for COVID-19. Close collaboration and communication between countries and institutions are conducive to eliminating academic barriers and further developing research related to the COVID-19 RNA vaccines.

From the perspective of the author, SAHIN U, SHI PY, LIU Y, TÜRECI Ö, and LEE J published the most articles. Professor Uğur Şahin, who had the highest number of total citations, had published 14 papers, 9 of which were concerned with the immunogenicity and effectiveness of COVID-19 mRNA vaccines, and pointed out that BNT162b2 has neutralizing activity on different COVID-19 variants. They also found that BNT162B2 can elicit the response of TH1 cells and antibodies. In addition, the safety of these vaccines has also been proved (37–46). Pei-Yong Shi, whose h-index was second only to Uğur Şahin, has published 16 articles during these four years, most of which pointed out the safety and immunogenicity of COVID-19 RNA vaccines. These vaccines can induce the persistent response of the human germinal center. He also found that some SARS-CoV-2 variants resist these RNA vaccines (25, 27, 37, 39, 47–56).

Most of the research on COVID-19 RNA vaccines was published in Vaccines (IF=7.8, Q1), indicating it is currently the most productive journal in this research field. Among the journals, the journal with the highest impact factor is the New England Journal of Medicine (IF=158.5, Q1), followed by Nature (IF=64.8, Q1). As for the co-cited journals, we could find that most of them are high-impact Q1 journals. These journals are high-quality international journals providing support for the study of COVID-19 RNA vaccines.

The top 20 most cited articles were mainly published between 2020 and 2021, and all seven were published in the New England Journal of Medicine, indicating the influence of the New England Journal of Medicine in this regard. In addition, the first four articles are all about the safety and effectiveness of the COVID-19 RNA vaccines. It can be seen that the safety and effectiveness of RNA vaccines have always been a hot topic in the discussion of the COVID-19 RNA vaccines.

Vaccines, Frontiers in Immunology, and Virus Basel are the journals that publish the most articles about the COVID-19 RNA vaccines. However, regarding influence, the New England Journal of Medicine has the highest h-index, m-index, and total citations, proving that it currently has the most significant influence in this field. Frontiers in Immunology has the highest g-index, proving its importance in the field of COVID-19 RNA vaccines. Frontiers in Immunology, Vaccines, and Journal of Medical Virology were the top three most influential journals in this field, which may be listed in the journal consideration for the relevant researchers.

According to the keywords, COVID-19, SAR-COV-2, and vaccine are currently the most concerning topics conducive to further research. The research hotspots in this field mainly include COVID-19, SAR-COV-2, and vaccine. We hope this work can provide new ideas for promoting scientific research and clinical applications of COVID-19 RNA vaccines.

In general, this study is the first comprehensive analysis that summarizes the research of the COVID-19 RNA vaccines using literature metrology methods. Our research findings provide valuable information for researchers in this field to understand the basic knowledge landscape, current research hotspots, and future opportunities and identify potential collaborators in the future.

The wide application of the COVID-19 RNA vaccines provides a good platform for the development of RNA vaccine, not only contributes to the research and development of COVID-19 RNA vaccines but also proves the effectiveness and safety of RNA vaccine to a certain extent and provides sufficient theoretical and technical support for the future application of RNA vaccine in other fields, such as cancer treatment.

### Limitations

Firstly, to ensure high-quality bibliometric analysis, the analysis of this study is based on articles in the Web of Science database, one of the most commonly used scientific publication databases. However, some studies may be omitted as they are published in non-SCI journals or other databases. Secondly, bibliometric analyses cannot completely replace system retrieval. Third, metrology cannot evaluate the quality of a single study because the citation index is time-dependent, meaning that recent articles may be less cited than earlier, even if they are more valuable. These limitations may slightly impact the overall results but are unlikely to alter the main trends presented in this article. In general, our research has provided a basis for understanding the research topics of the COVID-19 RNA vaccines and the production and application of the RNA vaccine.

## Methods

### Eligibility criteria and data source

In this study, research articles on RNA vaccines for COVID-19 published between 2020 and 2023 as original articles or reviews in English were considered eligible. Web of Science core collection database was used.

### Search strategy

In the advanced search option of the Web of Science database, using an appropriate combination of Boolean and wildcard search operators, the following keywords were searched: “Corona Virus Disease 2019”, “COVID-19”, “RNA”, and “vaccines”. The search was performed on January 11th, 2024, and the entire search strategy is presented in TS = ((RNA vaccine AND COVID-19) OR (RNA vaccine AND SARS-COV-2, the type of documents is set to “articles” and “reviews”. The language of articles is set as English only. Then, all the resulted information, including full records and cited references, was downloaded in txt format.

### Bibliometric analyses

Data management and bibliometric analyses were conducted using the Bibliometrix package (version 3.1.4) (57) and Biblioshiny (57) web apps under R (version 4.0.2). We retrieved all the main information and features included in the study. Publications and citation trends were constructed over four years. From 2020 to 2023, the most influential countries on COVID-19 RNA vaccine research were retrieved and presented as a cluster collaboration network. The cooperative world map represents world research cooperation, with the minimum edge set at 10. In addition, we identified the most productive institutions based on the highest number of paper contributions to the topic over the past four years. We used leading eigenvalue clustering algorithms to construct a collaborative network between institutions with more than five citations. We determined the author with the highest contribution based on the highest number of papers and the top 20 co-citation networks of influential authors. The 20 most cited references and the most influential journals were also identified, and some characteristics were searched, such as h-index, g-index, m-index, the total number of citations, the number of papers on the subject published in the journal, and the year when the journal began to publish COVID-19 RNA vaccine-related topics. In order to observe the inflow and outflow of journals, authors, and affiliated institutions that have contributed to the COVID-19 RNA vaccines in the past four years, a three-field plot was constructed. A tree chart was prepared to display keywords published on this topic from 2020 to 2023.

## Conclusions

RNA vaccine has essential research value and application prospects in COVID-19. The rapid increase in the number of publications shows that the research on the RNA vaccine for COVID-19 has attracted more attention from scholars worldwide. The main countries are the United States and China. However, cooperation and communication between countries and institutions still need to be strengthened. On the one hand, studying the immunogenicity and safety of RNA vaccines will help us to prevent COVID-19 variants infection and reduce vaccine side effects (58). On the other hand, compared with traditional vaccines, RNA vaccines have significant advantages in preventing COVID-19. Therefore, the study of COVID-19 RNA vaccines has essential application value in preventing COVID-19 infection and alleviating symptoms in the future (59). In addition to the related prevention research of COVID-19, attention can also be paid to the transformation of research achievements, that is, the clinical application of RNA vaccines in other diseases (58).

## Data availability statement

The original contributions presented in the study are included in the article/supplementary material. Further inquiries can be directed to the corresponding authors.

## Author contributions

ZC: Conceptualization, Visualization, Writing – original draft. ZL: Software, Validation, Writing – review & editing. YF: Writing – review & editing. AS: Writing – original draft. LW: Writing – review & editing. YS: Conceptualization, Funding acquisition, Supervision, Writing – original draft. CL: Conceptualization, Software, Supervision, Writing – original draft, Writing – review & editing.

## References

[1] Center JHUCR. COVID-19 dashboard by the Center for Systems Science and Engineering (CSSE) at Johns Hopkins University. Available online at: https://coronavirus.jhu.edu/map.html2023.

[2] Seo SH Jang Y. Cold-adapted live attenuated SARS-cov-2 vaccine completely protects human ACE2 transgenic mice from SARS-cov-2 infection. Vaccines (2020) 8(4):584. doi: 10.1101/2020.08.04.235689 33022950 PMC7712048

[3] Trimpert J Dietert K Firsching TC Ebert N Thi Nhu Thao T Vladimirova D. Development of safe and highly protective live-attenuated SARS-CoV-2 vaccine candidates by genome recoding. Cell Rep (2021) 36:109493. doi: 10.1016/j.celrep.2021.109493 34320400 PMC8289629

[4] Xia S Duan K Zhang Y Zhao D Zhang H Xie Z. Effect of an inactivated vaccine against SARS-coV-2 on safety and immunogenicity outcomes: interim analysis of 2 randomized clinical trials. Jama (2020) 324:951–60. doi: 10.1001/jama.2020.15543 PMC7426884 32789505

[5] Guebre-Xabier M Patel N Tian JH Zhou B Maciejewski S Lam K. NVX-CoV2373 vaccine protects cynomolgus macaque upper and lower airways against SARS-CoV-2 challenge. Vaccine (2020) 38:7892–6. doi: 10.1016/j.vaccine.2020.10.064 PMC7584426 33139139

[6] Wu Y Huang X Yuan L Wang S Zhang Y Xiong H. A recombinant spike protein subunit vaccine confers protective immunity against SARS-CoV-2 infection and transmission in hamsters. Sci Trans Med (2021) 13(606):eabg1143. doi: 10.1126/scitranslmed.abg1143 PMC9836081 34285130

[7] Yang S Li Y Dai L Wang J He P Li C. Safety and immunogenicity of a recombinant tandem-repeat dimeric RBD-based protein subunit vaccine (ZF2001) against COVID-19 in adults: two randomised, double-blind, placebo-controlled, phase 1 and 2 trials. Lancet Infect Dis (2021) 21:1107–19. doi: 10.1016/S1473-3099(21)00127-4 PMC7990482 33773111

[8] Xia S Zhang Y Wang Y Wang H Yang Y Gao GF. Safety and immunogenicity of an inactivated SARS-CoV-2 vaccine, BBIBP-CorV: a randomised, double-blind, placebo-controlled, phase 1/2 trial. Lancet Infect Dis (2021) 21:39–51. doi: 10.1016/S1473-3099(20)30831-8 33069281 PMC7561304

[9] Ella R Vadrevu KM Jogdand H Prasad S Reddy S Sarangi V. Safety and immunogenicity of an inactivated SARS-CoV-2 vaccine, BBV152: a double-blind, randomised, phase 1 trial. Lancet Infect Dis (2021) 21:637–46. doi: 10.1016/S1473-3099(20)30942-7 PMC7825810 33485468

[10] Kremsner PG Mann P Kroidl A Leroux-Roels I Schindler C Gabor JJ. Safety and immunogenicity of an mRNA-lipid nanoparticle vaccine candidate against SARS-CoV-2: A phase 1 randomized clinical trial. Wiener klinische Wochenschrift (2021) 133:931–41. doi: 10.1007/s00508-021-01922-y PMC8354521 34378087

[11] Zhang Y Zeng G Pan H Li C Hu Y Chu K. Safety, tolerability, and immunogenicity of an inactivated SARS-CoV-2 vaccine in healthy adults aged 18-59 years: a randomised, double-blind, placebo-controlled, phase 1/2 clinical trial. Lancet Infect Dis (2021) 21:181–92. doi: 10.1016/S1473-3099(20)30843-4 PMC7832443 33217362

[12] Corbett KS Edwards DK Leist SR Abiona OM Boyoglu-Barnum S Gillespie RA. SARS-CoV-2 mRNA vaccine design enabled by prototype pathogen preparedness. Nature (2020) 586:567–71. doi: 10.1038/s41586-020-2622-0 PMC7581537 32756549

[13] Wang Y Yang C Song Y Coleman JR Stawowczyk M Tafrova J. Scalable live-attenuated SARS-CoV-2 vaccine candidate demonstrates preclinical safety and efficacy. Proc Natl Acad Sci USA (2021) 118(29):e2102775118. doi: 10.1073/pnas.2102775118 34193524 PMC8307828

[14] Dai L Zheng T Xu K Han Y Xu L Huang E. A universal design of betacoronavirus vaccines against COVID-19, MERS, and SARS. Cell (2020) 182:722–33.e11. doi: 10.1016/j.cell.2020.06.035 32645327 PMC7321023

[15] Yang J Wang W Chen Z Lu S Yang F Bi Z. A vaccine targeting the RBD of the S protein of SARS-CoV-2 induces protective immunity. Nature (2020) 586:572–7. doi: 10.1038/s41586-020-2599-8 32726802

[16] Prize TN. Press Release: The Nobel Assembly at Karolinska Institutet. Available at: https://www.nobelprize.org/prizes/medicine/2023/press-release/2023.

[17] Pilkington EH Suys EJA Trevaskis NL Wheatley AK Zukancic D Algarni A. From influenza to COVID-19: Lipid nanoparticle mRNA vaccines at the frontiers of infectious diseases. Acta biomaterialia (2021) 131:16–40. doi: 10.1016/j.actbio.2021.06.023 34153512 PMC8272596

[18] Miao L Zhang Y Huang L. mRNA vaccine for cancer immunotherapy. Mol Cancer (2021) 20:41. doi: 10.1186/s12943-021-01335-5 33632261 PMC7905014

[19] Zhou X Berglund P Rhodes G Parker SE Jondal M Liljeström P. Self-replicating Semliki Forest virus RNA as recombinant vaccine. Vaccine (1994) 12:1510–4. doi: 10.1016/0264-410X(94)90074-4 7879415

[20] Cagigi A Loré K. Immune responses induced by mRNA vaccination in mice, monkeys and humans. Vaccines. (2021) 9(1):61. doi: 10.3390/vaccines9010061. 33477534 PMC7831080

[21] Krammer F. SARS-CoV-2 vaccines in development. Nature (2020) 586:516–27. doi: 10.1038/s41586-020-2798-3 32967006

[22] Cobb M. Who discovered messenger RNA? Curr biol: CB (2015) 25:R526–32. doi: 10.1016/j.cub.2015.05.032 26126273

[23] Thomas SJ Moreira ED Jr. Kitchin N Absalon J Gurtman A Lockhart S. Safety and Efficacy of the BNT162b2 mRNA Covid-19 Vaccine through 6 Months. N Engl J Med (2021) 385:1761–73. doi: 10.1056/NEJMoa2110345 PMC8461570 34525277

[24] Polack FP Thomas SJ Kitchin N Absalon J Gurtman A Lockhart S. Safety and efficacy of the BNT162b2 mRNA covid-19 vaccine. N Engl J Med (2020) 383:2603–15. doi: 10.1056/NEJMoa2034577 PMC7745181 33301246

[25] Mulligan MJ Lyke KE Kitchin N Absalon J Gurtman A Lockhart S. Phase I/II study of COVID-19 RNA vaccine BNT162b1 in adults. Nature (2020) 586:589–93. doi: 10.1038/s41586-020-2639-4 32785213

[26] Li J Hui A Zhang X Yang Y Tang R Ye H. Safety and immunogenicity of the SARS-CoV-2 BNT162b1 mRNA vaccine in younger and older Chinese adults: a randomized, placebo-controlled, double-blind phase 1 study. Nat Med (2021) 27:1062–70. doi: 10.1038/s41591-021-01330-9 33888900

[27] Walsh EE Frenck RW Jr. Falsey AR Kitchin N Absalon J Gurtman A. Safety and immunogenicity of two RNA-based covid-19 vaccine candidates. N Engl J Med (2020) 383:2439–50. doi: 10.1056/NEJMoa2027906 PMC7583697 33053279

[28] Pimpinelli F Marchesi F Piaggio G Giannarelli D Papa E Falcucci P. Fifth-week immunogenicity and safety of anti-SARS-CoV-2 BNT162b2 vaccine in patients with multiple myeloma and myeloproliferative Malignancies on active treatment: preliminary data from a single institution. J Hematol Oncol (2021) 14:81. doi: 10.1186/s13045-021-01090-6 34001183 PMC8128283

[29] Sebastian M Papachristofilou A Weiss C Früh M Cathomas R Hilbe W. Phase Ib study evaluating a self-adjuvanted mRNA cancer vaccine (RNActive^®^) combined with local radiation as consolidation and maintenance treatment for patients with stage IV non-small cell lung cancer. BMC Cancer (2014) 14:748. doi: 10.1186/1471-2407-14-748 25288198 PMC4195907

[30] Verbeke R Lentacker I De Smedt SC Dewitte H. Three decades of messenger RNA vaccine development. Nano Today (2019) 28:17. doi: 10.1016/j.nantod.2019.100766

[31] Xu S Yang K Li R Zhang L. mRNA vaccine era-mechanisms, drug platform and clinical prospection. Int J Mol Sci (2020) 21(18):6582. doi: 10.3390/ijms21186582 32916818 PMC7554980

[32] Iavarone C O’Hagan DT Yu D Delahaye NF Ulmer JB. Mechanism of action of mRNA-based vaccines. Expert Rev Vaccines (2017) 16:871–81. doi: 10.1080/14760584.2017.1355245 28701102

[33] Pardi N Hogan MJ Weissman D. Recent advances in mRNA vaccine technology. Curr Opin Immunol (2020) 65:14–20. doi: 10.1016/j.coi.2020.01.008 32244193

[34] Wrapp D Wang NS Corbett KS Goldsmith JA Hsieh CL Abiona O. Cryo-EM structure of the 2019-nCoV spike in the prefusion conformation. Sci (New York NY) (2020) 367:1260. doi: 10.1126/science.abb2507 PMC7164637 32075877

[35] Hoffmann M Kleine-Weber H Schroeder S Krueger N Herrler T Erichsen S. SARS-coV-2 cell entry depends on ACE2 and TMPRSS2 and is blocked by a clinically proven protease inhibitor. Cell (2020) 181:271. doi: 10.1016/j.cell.2020.02.052 32142651 PMC7102627

[36] Baden LR El Sahly HM Essink B Kotloff K Frey S Novak R. Efficacy and safety of the mRNA-1273 SARS-coV-2 vaccine. N Engl J Med. (2021) 384:403–16. doi: 10.1056/NEJMoa2035389. PMC7787219 33378609

[37] Vogel AB Kanevsky I Che Y Swanson KA Muik A Vormehr M. BNT162b vaccines protect rhesus macaques from SARS-CoV-2. Nature (2021) 592:283–9. doi: 10.1038/s41586-021-03275-y 33524990

[38] Sahin U Muik A Vogler I Derhovanessian E Kranz LM Vormehr M. BNT162b2 vaccine induces neutralizing antibodies and poly-specific T cells in humans. Nature (2021) 595:572–7. doi: 10.1038/s41586-021-03653-6 34044428

[39] Sahin U Muik A Derhovanessian E Vogler I Kranz LM Vormehr M. COVID-19 vaccine BNT162b1 elicits human antibody and T(H)1 T cell responses. Nature (2020) 586:594–9. doi: 10.1038/s41586-020-2814-7 32998157

[40] Quandt J Muik A Salisch N Lui BG Lutz S Krüger K. Omicron BA.1 breakthrough infection drives cross-variant neutralization and memory B cell formation against conserved epitopes. Sci Immunol (2022) 7:eabq2427. doi: 10.1126/sciimmunol.abq2427 35653438 PMC9162083

[41] Muik A Wallisch AK Sänger B Swanson KA Mühl J Chen W. Neutralization of SARS-CoV-2 lineage B.1.1.7 pseudovirus by BNT162b2 vaccine-elicited human sera. Sci (New York NY) (2021) 371:1152–3. doi: 10.1126/science.abg6105 PMC7971771 33514629

[42] Muik A Lui BG Wallisch AK Bacher M Mühl J Reinholz J. Neutralization of SARS-CoV-2 Omicron by BNT162b2 mRNA vaccine-elicited human sera. Sci (New York NY) (2022) 375:678–80. doi: 10.1126/science.abn7591 PMC9836206 35040667

[43] Muik A Lui BG Quandt J Diao H Fu Y Bacher M. Progressive loss of conserved spike protein neutralizing antibody sites in Omicron sublineages is balanced by preserved T cell immunity. Cell Rep (2023) 42:112888. doi: 10.1016/j.celrep.2023.112888 37527039

[44] Muik A Lui BG Bacher M Wallisch AK Toker A Finlayson A. Omicron BA.2 breakthrough infection enhances cross-neutralization of BA.2.12.1 and BA.4/BA.5. Sci Immunol (2022) 7:eade2283. doi: 10.1126/sciimmunol.ade2283 36125366 PMC9529054

[45] Muik A Lui BG Bacher M Wallisch AK Toker A Couto CIC. Exposure to BA.4/5 S protein drives neutralization of Omicron BA.1, BA.2, BA.2.12.1, and BA.4/5 in vaccine-experienced humans and mice. Sci Immunol (2022) 7:eade9888. doi: 10.1126/sciimmunol.ade9888 36378074 PMC9765452

[46] Rohde CM Lindemann C Giovanelli M Sellers RS Diekmann J Choudhary S. Toxicological assessments of a pandemic COVID-19 vaccine-demonstrating the suitability of a platform approach for mRNA vaccines. Vaccines (2023) 11(2):417. doi: 10.3390/vaccines11020417 36851293 PMC9965811

[47] Chen RE Zhang X Case JB Winkler ES Liu Y VanBlargan LA. Resistance of SARS-CoV-2 variants to neutralization by monoclonal and serum-derived polyclonal antibodies. Nat Med (2021) 27:717–26. doi: 10.1038/s41591-021-01294-w PMC8058618 33664494

[48] Hajnik RL Plante JA Liang Y Alameh MG Tang J Bonam SR. Dual spike and nucleocapsid mRNA vaccination confer protection against SARS-CoV-2 Omicron and Delta variants in preclinical models. Sci Trans Med (2022) 14:eabq1945. doi: 10.1126/scitranslmed.abq1945 PMC9926941 36103514

[49] Liu J Liu Y Xia H Zou J Weaver SC Swanson KA. BNT162b2-elicited neutralization of B.1.617 and other SARS-CoV-2 variants. Nature (2021) 596:273–5. doi: 10.1038/s41586-021-03693-y 34111888

[50] Liu Y Liu J Xia H Zhang X Fontes-Garfias CR Swanson KA. Neutralizing activity of BNT162b2-elicited serum. N Engl J Med (2021) 384:1466–8. doi: 10.1056/NEJMc2102017 PMC7944950 33684280

[51] Pegu A O’Connell SE Schmidt SD O’Dell S Talana CA Lai L. Durability of mRNA-1273 vaccine-induced antibodies against SARS-CoV-2 variants. Sci (New York NY) (2021) 373:1372–7. doi: 10.1101/2021.05.13.444010 PMC8691522 34385356

[52] Schmitz AJ Turner JS Liu Z Zhou JQ Aziati ID Chen RE. A vaccine-induced public antibody protects against SARS-CoV-2 and emerging variants. Immunity. (2021) 54:2159–66.e6 doi: 10.1016/j.immuni.2021.08.013 34464596 PMC8367776

[53] Turner JS O’Halloran JA Kalaidina E Kim W Schmitz AJ Zhou JQ. SARS-CoV-2 mRNA vaccines induce persistent human germinal centre responses. Nature (2021) 596:109–13. doi: 10.1038/s41586-021-03738-2 PMC8935394 34182569

[54] Wang L Kainulainen MH Jiang N Di H Bonenfant G Mills L. Differential neutralization and inhibition of SARS-CoV-2 variants by antibodies elicited by COVID-19 mRNA vaccines. Nat Commun (2022) 13:4350. doi: 10.1038/s41467-022-31929-6 35896523 PMC9328008

[55] Xia H Zou J Kurhade C Cai H Yang Q Cutler M. Neutralization and durability of 2 or 3 doses of the BNT162b2 vaccine against Omicron SARS-CoV-2. Cell Host Microbe (2022) 30:485–8.e3. doi: 10.1016/j.chom.2022.02.015 35245438 PMC8853806

[56] Xie X Zou J Kurhade C Liu M Ren P Pei-Yong S. Neutralization of SARS-CoV-2 Omicron sublineages by 4 doses of the original mRNA vaccine. Cell Rep (2022) 41:111729. doi: 10.1016/j.celrep.2022.111729 36402138 PMC9647030

[57] Aria M Cuccurullo C. bibliometrix: An R-tool for comprehensive science mapping analysis. J Informetrics (2017) 11:959–75. doi: 10.1016/j.joi.2017.08.007.

[58] Goel RR Painter MM Apostolidis SA Mathew D Meng W Rosenfeld AM. mRNA vaccines induce durable immune memory to SARS-CoV-2 and variants of concern. Sci (New York NY) (2021) 374:abm0829. doi: 10.1126/science.abm0829 PMC9284784 34648302

[59] Andrews N Stowe J Kirsebom F Toffa S Sachdeva R Gower C. Effectiveness of COVID-19 booster vaccines against COVID-19-related symptoms, hospitalization and death in England. Nat Med (2022) 28:831–7. doi: 10.1038/s41591-022-01699-1 PMC9018410 35045566

